# Evaluation of the learning curve of pediatric kidney transplantation anesthesia

**DOI:** 10.3906/sag-2012-291

**Published:** 2021-06-28

**Authors:** Tümay ULUDAĞ YANARAL, Pelin KARAASLAN

**Affiliations:** 1 Department of Anesthesiology and Reanimation, Faculty of Medicine, İstanbul Medipol University, İstanbul Turkey

**Keywords:** Anesthesia, chronic kidney failure, kidney transplantation, learning curve, pediatrics

## Abstract

**Background/aim:**

Pediatric kidney transplantation (PKT) anesthesia brings some different challenges than adult kidney transplantation (KT) anesthesia and there are still no studies analyzing the role of experience on PKT outcomes. In this study, we aimed to evaluate the anesthesia learning curve in pediatric kidney transplants performed in our institution and the effect of increasing experience on renal transplantation-related data.

**Materials and methods:**

Patients age ≤ 18 years who underwent KT were included in the study, while patients age >18 years were excluded. Patients were divided into 3 groups according to the date of transplant, as the first 10 patients in Group 1, the second 10 patients in Group 2, and the final 11 in Group 3. Groups were compared according to recorded data.

**Results:**

Thirty-one patients were included in the study. Age, sex, and body mass index were matched between the 3 groups. The mean durations of dialysis were 75.0 ± 63.0, 22.4 ± 27.9, and 5.7 ± 4.5 months for Group 1, Group 2, and Group 3, respectively (p = 0.009). Blood loss, duration of postoperative mechanical ventilation, and length of stay in the intensive care unit (ICU) were comparable between the groups. The duration of anesthesia gradually shortened from Group 1 to Group 3 but there was no significant difference between the groups. The mean number of red blood cell (RBC) transfusion was 0.9 ± 0.7 unit in group 1. It decreased to a mean of 0.6 ± 0.7 unit for group 2, and afterward significant decrease occurred down to 0 for group 3 (p = 0.004).

**Conclusion:**

Our results demonstrate that considering the decrease in preoperative dialysis duration and operative RBC transfusion, 20 patients may be enough for anesthesia competency. Transplantation anesthesia experience before PKT, anesthesia technique, and patient characteristics may differ between institutions. Therefore, further prospective studies with established learning curve goals, larger patient volumes, and more variables are needed to validate our results.

## 1. Introduction

End stage renal disease (ESRD) is the terminal stage of chronic kidney disease and the management options are hemodialysis, peritoneal dialysis or transplantation. Kidney transplantation (KT) is the most effective treatment modality in patients with ESRD. Over the past decade, a significant increase in survival rates after KT has been observed due to improvements in immunosuppressive medication, organ procurement, patient preparation, anesthesia, and surgical techniques. Consequently, there has been an increase in the number of kidney transplants performed [1,2].

ESRD is a rare condition in pediatric age group. In Europe, pediatric kidney transplantation (PKT) accounts for only about 4% of all transplantations performed [3]. In pediatric patients, KT can cause significant changes in the patient’s hemodynamics due to pre-existing chronic diseases, a large incision, and placement of a relatively big donor kidney. All these factors increase the complexity of anesthesia management. The outcomes of a transplantation can be influenced by various factors including anesthesia, surgical skills and experience, standardization of the procedure, and the quality of training. 

The learning curve is a representation of the process by which a physician develops skills in the training period, and the initial inexperience at the beginning of the curve can lead to a lower success rate and longer operating time. PKT anesthesia brings some different challenges than adult KT anesthesia and there are still no studies analyzing the role of experience on PKT outcomes. In this study, we aimed to evaluate the anesthesia learning curve in pediatric kidney transplants performed in our institution and the effect of increasing experience on renal transplantation-related data.

## 2. Materials and methods

### 2.1. Study population

After obtaining local ethics committee approval (approval number: 2020/928), the medical records of children under the age 18 and who underwent kidney transplantation in our institute between March 2014 and November 2020 were retrospectively analyzed. Data were collected using clinical follow-up forms and hospital information management system. Patient’s characteristics, chronic kidney disease related parameters, anesthesia and surgery related data were recorded. The only criterion for inclusion and exclusion was age, and all patients age ≤ 18 years who underwent KT were included in the study, while patients age >18 years were excluded. Patients were divided into 3 groups according to the date of transplant, as the first 10 patients in Group 1, the second 10 patients in Group 2, and the final 11 in Group 3. Groups were compared according to preoperative characteristic, operative data, and postoperative outcomes.

### 2.2. Anesthesia technique

While the surgical team had experience in PKT at the beginning of the study, the anesthesia team and other staff had no experience in PKT. Anesthesia procedures of all patients were performed by the same anesthesiology team, which had a previous experience with at least 30 adult KT cases. The team consisted of 2 anesthesiologists. One of them observed 5 PKT procedures at a transplantation center with experience in PKT and led the team. All patients were evaluated in detail during the preoperative anesthesia visit. KT of all patients were performed under general anesthesia. Following hemodynamic monitoring, induction of general anesthesia was applied with propofol (2–3 mg/kg), fentanyl (1 μg/kg), and atracurium besylate (0.5 mg/kg) by intravenous cannula, preserving the arteriovenous fistula, if any. After endotracheal intubation, mechanical ventilation was applied in a volume-controlled mode with an end-tidal CO2 value 30–35 mmHg. Sevoflurane (2%–3%) in a mixture of 40% oxygen and 60% air was used for anesthesia maintenance. An ultrasound-guided central venous catheter was placed in the internal jugular vein for fluid infusion, central venous pressure (CVP), and mixed venous oxygen saturation monitoring. A CVP of 10–15 cm H2O was maintained. 5% dextrose and other crystalloids were used for fluid maintenance. An arterial cannula was placed in the radial artery for direct blood pressure monitoring and arterial blood gas analysis. Atracurium besylate (0.1 mg/kg) was administered at routine intervals to maintain neuromuscular block. For the maintenance of analgesia remifentanil (0.25 µg/kg/min) was infused. Before renal reperfusion steroid (10 mg/kg) and furosemide (1–2 mg/kg) were administered. After graft reperfusion, renal artery flow was checked with intraoperative Doppler ultrasound. At the end of surgery, all patients were transferred to the intensive care unit (ICU) for close follow-up and uninterrupted management.

### 2.3. Statistical analysis

Normal distribution of data was checked by Shapiro–Wilk test. A one-way ANOVA was used to compare the means of the normally distributed data. If the data were not normally distributed, the Kruskal–Wallis test was used. Chi-square and Fisher’s exact tests were used to compare categorical variables. Post-hoc analysis was performed with Tukey’s and Games–Howell tests for comparing data that statistically significant difference between groups. P value was considered significant if less than 0.05. The Statistical Package for the Social Sciences v. 22.0 (IBM Corp., Armonk, NY, USA) was used to analyze the data.

## 3. Results

Thirty-one patients were included in the study. Age, sex, body mass index, hemodialysis, and peritoneal dialysis rates were matched between the 3 groups (p = 0.930, p = 0.452, p = 0.301, p = 0.220 and p = 0.717, respectively). The mean durations of dialysis were 75.0 ± 63.0, 22.4 ± 27.9 and 5.7 ± 4.5 months for Group 1, Group 2, and Group 3, respectively (p = 0.009). Demographic and clinical characteristics of patients are depicted in Table 1.

**Table 1:** Demographic and clinical characteristics of the patients, and their comparison between the groups.

	Total	Group 1	Group 2	Group 3	p
n	31	10	10	11	
Age (years)*	12.6 ± 4.6	12.7 ± 5.2	12.5 ± 4.2	12.6 ± 4.9	0.930
Sex (male/female)	15/16	4/6	4/6	7/4	0.452
Weight (kg)*	36.2 ± 18.2	33.2 ± 16.7	38.1 ± 16.6	37.3 ± 22.1	0.820
Height (cm)*	138.6 ± 24.3	134.6 ± 25.3	139.3 ± 21.9	141.6 ± 27.1	0.808
BMI (kg/m2)*	17.5 ± 3.8	17.1 ± 3.5	18.6 ± 3.6	17.1 ± 4.6	0.301
Hemodialysis	19 (61.2%)	5 (50%)	5 (50%)	9 (81.8%)	0.220
Peritoneal dialysis	4 (12.9%)	2 (20%)	1 (10%)	1 (9.1%)	0.717
Duration of dialysis (months)*	28.2 ± 42.9	75.0 ± 63.0	22.4 ± 27.9	5.7 ± 4.5	0.009
Hypertension	15 (48.3%)	3 (30%)	6 (60%)	6 (54.5%)	0.357
Diabetes mellitus	1 (3.2%)	0 (0%)	0 (0%)	1 (9.1%)	0.391

*: mean ± standard deviation. BMI, body mass index.

The graft was living donor kidney in 29 patients and deceased donor kidney in 2 patients. Blood loss, duration of postoperative mechanical ventilation, and length of stay in the ICU were comparable between the groups (p = 0.794, p = 0.741, p = 0.971, respectively). Also, postoperative daily urine output was similar for the 3 groups. The duration of anesthesia gradually shortened from Group 1 to Group 3 but there was no significant difference between the groups (234.5 ± 17.6, 219.6 ± 20.2, and 208.4 ± 16.9 min, respectively, p = 0.121). Similarly, we found a trend towards a decrease in the duration of surgery from Group 1 to Group 3, but it was not statistically significant between the groups (179.0, 172.0, and 167.7 min, respectively, p = 0.136). The mean number of red blood cell (RBC) transfusion was 0.9 ± 0.7 unit in Group 1. It decreased to a mean of 0.6 ± 0.7 unit for Group 2, and following thi significant decrease occurred down to 0 for Group 3 (p = 0.004). However, fresh-frozen plasma transfusion was similar between groups (Table 2).

**Table 2:** Comparison of surgery-related parameters.

	Group 1 (n = 10)	Group 2 (n = 10)	Group 3 (n = 11)	p
Kidney donor (deceased/living)	1/9	1/9	0/11	0.555
Duration of anesthesia (min)*	234.5 ± 17.6	219.6 ± 20.2	208.4 ± 16.9	0.121
Duration of surgery (min)*	179.0 ± 14.5	172.0 ± 19.9	167.7 ± 17.5	0.136
Blood loss (mL)*	182.5 ± 78.2	167.5 ± 44.1	165.9 ± 55.0	0.794
Red blood cell suspension transfusion (unit)*	0.9 ± 0.7	0.6 ± 0.7	0	0.004
Fresh-frozen plasma transfusion (unit)*	0.5 ± 0.8	0.1 ± 0.3	0	0.112
Postoperative first 24 h urine output (mL)*	6956.7 ± 3907.1	14791.2 ± 4677.4	6494.6 ± 1958.2	0.708
Postoperative second 24 h urine output (mL)*	6065.6 ± 3288.4	5121.5 ± 2538.3	8181.3 ± 5234.4	0.435
Postoperative third 24 h urine output (mL)*	4854.5 ± 1977.9	4441.0 ± 1936.7	5621.8 ± 2332.0	0.676
Postoperative mechanical ventilation	2 (20%)	1 (10%)	2 (18.2%)	0.809
Duration of postoperative mechanical ventilation (h)*	4.0 ± 11.3	1.2 ± 3.8	2.5 ± 7.2	0.741
Length of stay in ICU (days)*	1.4 ± 0.9	1.9 ± 2.5	1.2 ± 0.4	0.971
Length of stay in hospital (days)*	10.5 ± 6	15.8 ± 21.7	9.7 ± 2.1	0.328

*: mean ± standard deviation; ICU: intensive care unit.

Preoperative and postoperative creatinine, hemoglobin, sodium, and potassium were comparable for the 3 groups (Table 3). Post-hoc analysis was performed to compare the groups separately, according to variables that showed statistically significant differences (Table 4). Although there was no difference in the duration of dialysis between Group 1 and Group 2 and between Group 2 and Group 3. Group 3 had shorter duration of dialysis than group 1 (p = 0.017). The number of RBC transfusions in group 3 was significantly lower than group 1 (p = 0.004). Group 1 and group 2, and group 2 and group 3 were comparable by the number of RBC transfusions.

**Table 3:** Comparison of laboratory parameters.

	Group 1 (n = 10)	Group 2 (n = 10)	Group 3 (n = 11)	p
Creatinine (mg/dL)*				
Preoperative	5.6 ± 2.1	6.4 ± 3.2	5.6 ± 1.7	0.715
Postoperative 1	1.8 ± 1.4	2.5 ± 1.8	1.9 ± 1.3	0.616
Postoperative 2	0.9 ± 0.7	1.4 ± 0.4	1.2 ± 0.8	0.649
Hemoglobin (g/dL)*				
Preoperative	9.7 ± 1.9	9.2 ± 1.3	9.5 ± 1.7	0.795
Postoperative	8.9 ± 1.4	9.0 ± 1.3	8.7 ± 1.1	0.858
Na (mmoL/L)*				
Preoperative	140.0 ± 4.6	138.7 ± 3.1	137.8 ± 2.0	0.389
Postoperative 1	138.8 ± 6.8	135.7 ± 2.6	136.9 ± 2.9	0.051
Postoperative 2	137.5 ± 4.6	136.3 ± 3.7	138.9 ± 2.4	0.214
K (mmoL/L)*				
Preoperative	4.9 ± 0.9	4.7 ± 0.4	4.6 ± 0.8	0.653
Postoperative 1	3.9 ± 0.5	4.4 ± 0.9	4.3 ± 0.5	0.302
Postoperative 2	4.4 ± 0.5	4.3 ± 0.8	3.8 ± 0.4	0.066

*: mean ± standard deviation. Na: sodium; K: potassium.

**Table 4:** Post-hoc analysis.

	Group 1 vs.Group 2	Group 1 vs.Group 3	Group 2 vs.Group 3
Duration of dialysis (months)	0.092	0.017	0.689
Red blood cell suspension transfusion (unit)	0.484	0.004	0.061

## 4. Discussion

It is critical to understand the number of procedures a physician must perform before reaching a level of safety and competence. Studies aimed at defining the learning curve in anesthetic procedures have been reported. For the learning curve of transversus abdominal plane block, Vial et al. reported that 20 procedures were necessary for residents to become competent [4]. Another study on the learning curve of residents for specific anesthesia procedures proposed that six-month rotation could not be sufficient for competence [5]. To the best of our knowledge, our study was the first to evaluate the learning curve on PKT with parameters such as anesthesia time, operation time, blood loss, blood transfusion rate, duration of mechanical ventilation, and postoperative outcomes.

The number of studies reporting the duration of anesthesia in PKT is limited and to our knowledge there are no studies comparing the anesthesia duration with experience. Voet et al. reported PKT anesthesia duration as 294 min in their study on children under 20 kg [6]. Yet, the duration of anesthesia is slightly shorter in adults. In a study comparing the general anesthesia and epidural plus general anesthesia in adult patients, anesthesia durations were 130 min and 153 min, respectively [7]. With the increasing experience, the anesthesia duration is expected to decrease. In this study, the duration of anesthesia between the groups reduced progressively. For the 21st to 31st patient, the duration of anesthesia was reduced down to 208.4 min. Although there was no statistically significant difference, the mean duration of anesthesia difference between 1st to 10th patients and 21st to 31st patients was 26.1 min. 

Other important clinical indicator of competence is the duration of surgery. In the study by Mehrabi et al. involving 540 patients, mean operation time was reported as 188 min [8]. In a recent study, Ahlawat et al. investigated the learning curve of robotic KT. The aforementioned study reported the robotic console time as 187 and 131 min for experienced and inexperienced physicians, respectively. Ahlawat et al. concluded that the learning curve of robotic KT is short and the skill could be achieved at the 20th to 25th case [9]. We demonstrated that the mean duration of surgery decreased from 179 min for the first 10 patients to 167.7 min for the 21st to 31st patients. Although there was no statistically significant difference, it was observed that surgery time decreases progressively for the 1st to 10th patients compared to the 21st to 31st patients. We have been performing PKT in our hospital for the last 6 years and our team had high adult kidney transplant experience before starting PKT. We attribute the similarity of our surgery time to the literature in the first 31 patients to our high adult transplantation experience.

Bleeding is a common problem during a major surgery and surgical technique is the most important factor in reducing blood loss. In 188 pediatric recipients, Odeh et al. reported an average blood loss of 173 mL [10]. In another study, the average blood loss was reported to be 125 mL in a clinic with 48 years of surgical experience [8]. Although our results are yet not comparably as low, we improved over time and the mean blood loss was 165 mL for the 21st–31st patients. However, the difference in the blood loss between the groups was not statistically significant. (p = 0.794). Maintenance of normovolemia, adequate ventilation, avoidance of hypothermia, and acidosis seem to be crucial in the management of blood loss.

In addition to being a major surgery, PKT has significant effects on blood volume because larger adult size graft retains more of the circulating blood volume. Anesthetic technique is very important in fluid management and transfusion of blood products. Over the years, we started to use Ringer’s lactate instead of normal saline in fluid management. We think that metabolic acidosis can be controlled more easily in this way. In our study, preoperative hemoglobin level and operative blood loss were similar for the study groups. However, there was a significant difference between the 3 groups for the number of RBC transfusions. We observed that RBC transfusion requirement decreases as experience increases (p = 0.004). In post-hoc analysis, it was found that this significant difference was present between the first 10 patients and 21st to 31st patients. After the first 20 patients, we have reached the experience to manage blood loss without RBC transfusion. Although the number of studies reporting results on transfusion is limited, our data correlates with the other published study in which an average of 1 unit of RBC transfusion was performed [10]. We recommend CVP monitoring and rapid treatment of hypotension with crystalloids to ensure adequate renal perfusion in case of bleeding.

All of our patients were transferred to ICU after transplantation. Management of fluid and electrolyte imbalance and close monitoring of hemodialysis need are the main purposes of postoperative care in the ICU. There is lack of data on postoperative ICU durations for pediatric recipients and the first study for adults was published in 1995 [11]. In that study, the mean duration of mechanical ventilation and the mean length of stay in the ICU were reported as 2.8 days and 5.1 days, respectively. Over the years, these durations have decreased with medical and technological advances and improvements in the learning curve. In the present study, the mean ICU length of stay (1.4 days), and the mean duration of mechanical ventilation (3 h) were shorter compared to the aforementioned study. In our cohort, the length of stay in the ICU was similar for the three groups and the increase in experience did not change the mean ICU length of stay and the mean duration of mechanical ventilation. However, nowadays, if the patient is hemodynamically stable, we have been trying extubation in the operating room.

After the ICU period, hospitalization is required for a while due to need for intravenous fluid, pain relievers, and bed rest. Parental expectations and physician experience are also influential in the length of hospital stay (LOS). Krell et al. investigated the factors affecting the long postoperative stay and concluded that variation in practice style was more attributable rather than complications or patient-related factors [12]. Dias et al. evaluated the enhanced recovery after surgery (ERAS) protocol for KT and they reported an average hospital stay of 5 and 7.5 days for the ERAS and control group, respectively [13]. The mean LOS in our study was longer (11 days). LOS was not different between the groups, and experience had no effect on LOS. However, LOS in our study was shorter compared to the study involving pediatric transplants after 1967, which reported a mean hospital stay of 25 days [8]. This long duration is probably due to the hospitalization of patients with delayed graft function in the past years. Our current practice strategy is to treat these patients at home as much as possible. 

Transplant candidates with end-stage renal disease require dialysis until a suitable donor kidney becomes available. It has been demonstrated that longer duration on dialysis has a negative impact on graft and patient survival [14,15]. Analysis of the European Society of Pediatric Nephrology showed that the best 5-year graft survival (90%) was among children who underwent preemptive transplantation or following short term dialysis (<1.5 months) [16]. Although the time from the initiation of dialysis to transplantation varies according to the graft type, it has been reported as 10 to 25 months [8,17]. The mean dialysis duration in our first 10 cases were much longer than those reported in the literature. However, our increasing experience in PKT significantly shortened the preoperative dialysis duration of transplant candidates. Post-hoc analysis revealed that this significant difference occurred after 20 patients (p = 0.017). Probably, not only the experience of anesthesia but also the increased experience of the surgical and transplant team is effective in this result.

The present study is the first to evaluate the learning curve of pediatric kidney transplant anesthesia. Yet, the study has its own limitations. Firstly, the study included relatively small patient volume with a retrospective nature. Secondly, although there were no intraoperative complications, postoperative complications were not evaluated. Finally, comparison could not be made due to the lack of similar studies. These limitations should be considered in later studies.

In conclusion, our results demonstrate that considering the decrease in preoperative dialysis duration and operative RBC transfusion, 20 patients may be enough for anesthesia competency. Transplantation anesthesia experience before PKT, anesthesia technique, and patient characteristics may differ between institutions. Therefore, further prospective studies with established learning curve goals, larger patient volumes, and more variables are needed to validate our results.

## Informed consent

This study was approved by the ethics committee of **İ** stanbul Medipol University (no.: 928), and written informed consent was obtained from the patient’s parents.
